# Editorial: Case reports in thrombosis: 2022

**DOI:** 10.3389/fcvm.2023.1219274

**Published:** 2023-05-22

**Authors:** Luca Spiezia

**Affiliations:** General Medicine and Thrombotic and Haemorrhagic Diseases Unit, Department of Medicine, University of Padova, Padova, Italy

**Keywords:** case report, antithrombin deficiency, dilated cardiomyopathy, bi-atrial thrombus, ulcerative colitis, Kounis syndrome, right atrial mass, vaccine-induced thrombotic thrombocytopenia

**Editorial on the Research Topic** Case reports in thrombosis: 2022

This editorial presents the collection of articles published in Frontiers in Cardiovascular Medicine: Case Reports in Thrombosis: 2022. The aim of this Research Topic was to highlight unique cases of patients with unexpected or atypical thrombosis, clinical course or treatment response. This collection comprises only Case Reports that are original and significantly advance the field, and namely: (i) rare cases with typical features; (ii) common cases with atypical features; (iii) cases with a convincing response to new treatments, i.e., single case of off-label use.

The following are the articles in the Frontiers in Cardiovascular Medicine: Case Reports in Thrombosis: 2022.

*Missense mutation of SERPINC1 (p.Ser426Leu) in a young patient presenting as refractory and recurrent venous thromboembolism: A case report*, by Yu et al. Inherited thrombophilia plays a crucial role in the pathophysiology of venous thromboembolism (1). This study found an association between resistance to heparin and inherited antithrombin deficiency stemming from a heterozygous missense mutation of the gene SERPINC1. A switch to warfarin and rivaroxaban proved effective. This article highlights that genetic testing may help identify specific mutations and guide clinicians in the choice of anticoagulant therapy.

*Case Report: A Mysterious Giant Thrombus in the Right Atrium in a Patient With Dilated Cardiomyopathy,* by Lian et al. A thrombus in the right atrium is an extremely rare and unlikely occurrence in patients with dilated cardiomyopathy (2). This study reports that further investigation revealed cancer-associated thrombosis. It is paramount to perform a thorough diagnostic workup to detect the presence of occult cancer in patients who present thrombi in unusual sites. Enoxaparin resulted in a partial resolution of the thrombus after 3 weeks.

*Case report: Bi-atrial thrombus after occlusion of atrial septal defect with acute cerebral infarction and pulmonary embolism,* by Xiong et al. This study focuses on a patient who developed acute cerebral infarction and pulmonary following transcatheter closure of atrial septal defect. Intracardiac devices are increasingly used in place of traditional thoracotomy (3). This study highlights the risk of long-term device-related complications, and the importance of both a multidisciplinary team and three-dimensional transesophageal echocardiography for the diagnosis and treatment of intracardiac device-related thrombus.

*Acute pulmonary embolism following corticosteroid administration in acute severe ulcerative colitis with gastrointestinal bleeding: A case report,* by Liu et al. Ulcerative colitis is a chronic illness often associated with gastrointestinal bleeding and venous thromboembolism (VTE). This article discusses a patient with ulcerative colitis who developed VTE following corticosteroids treatment, resulting in a hypercoagulable state, bilateral pulmonary embolism and poor prognosis. This study highlights the possible clinical impact of exogenous glucocorticoids on the risk of VTE (4) and that there is currently no consensus among experts on the optimal anticoagulation regimen in these patients.

*Case report: Cefoperazone-sulbactam induced Kounis syndrome and cardiogenic shock,* by Ding et al. Kounis syndrome is a life-threatening, allergic coronary artery disease induced by various drugs and environmental factors (5). This case report presents a patient who developed a severe allergic reaction to an infusion of cefoperazone-sulbactam for recurrent infections. An urgent coronary angiography revealed in-stent thrombosis and the diagnosis was type III Kounis syndrome with cardiogenic shock. This article highlights the importance of screening patients for drug allergies, especially those with a coronary artery stent and a history of cephalosporin allergy.

*Case report: A rare case of recurrent right atrial mass dramatically disappeared after anticoagulation,* by Wang et al. The presence of right heart thrombosis in patients with pulmonary embolism is very rare, albeit associated with a high mortality (6). Although echocardiography is often used, cardiac MRI is the imaging tool of choice to ascertain whether a right atrial mass is a myxoma or a thrombus. This study suggests that anticoagulation should be initiated as early as possible in patients who present with severe wheezing and extreme dyspnoea due to right atrial mass.

*A case report of vaccine-induced immune thrombotic thrombocytopenia (VITT) with genetic sequencing analysis,* by Mendes-de-Almeida et al. In recent years there have been increasing reports of a rare but life-threatening syndrome called vaccine-induced immune thrombocytopenia and thrombosis (VITT) after adenoviral vector vaccines, such as ChAdOx1 nCov-19 (7). This case report aimed to identify potential predisposing risk factors to VITT using genetic sequencing analysis, which revealed a benign rs1801133 homozygous variant in MTHFR and the rs116667976, rs2036914, and rs4253405 in the F11; and homozygous mutations in IFNAR2, IFNW1, TBK1, TICAM1, TLR3 genes without reported clinical significance. Although larger studies are needed to ascertain the causal nexus, identifying predisposing genetic variants may help clinicians detect early signs and symptoms of VITT and promptly initiate lifesaving treatment.
